# Supplementary material for: C3 glomerulonephritis associated with monoclonal gammopathy of renal significance: a diagnostic and therapeutic challenge
Source: J Bras Nefrol. 2024 Sep 16;46(4):e20240092. doi: 10.1590/2175-8239-JBN-2024-0092en (PMC11542637; doi:10.1590/2175-8239-JBN-2024-0092en)
Supplement: Translation

# Translation: Glomerulonefrite C3 associada a gamopatia monoclonal de significância renal: um desafio diagnóstico e terapêutico

**DOI:** 10.1590/2175-8239-JBN-2024-0092pt

**Authors:** Bárbara Beirão, Mariana Freitas, Natália Silva, Patrícia Ferraz, Catarina Prata, Teresa Morgado

**Affiliations:** 1 Centro Hospitalar de Trás-os-Montes e Alto Douro, Hospital de Vila Real, Serviço de Nefrologia, Vila Real, Portugal.; 2 Centro Hospitalar de Trás-os-Montes e Alto Douro, Hospital de Vila Real, Serviço de Hematologia Clínica, Vila Real, Portugal.

## Introdução

A gamopatia monoclonal de significância renal (GMSR) é o termo utilizado para descrever condições hematológicas nas quais a produção e secreção de uma imunoglobulina monoclonal (IgM) causa danos renais^1^. Esses distúrbios hematológicos não preenchem os critérios diagnósticos para mieloma múltiplo ou doença linfoproliferativa e, portanto, não preenchem os critérios hematológicos previamente definidos para o tratamento direcionado^1^.

O espectro de doenças renais associadas à GMSR é muito amplo, e a lesão renal pode ser causada diretamente pela deposição da proteína monoclonal ou, menos comumente, pela desregulação da via alternativa (VA) do complemento^1–3^. Nesse mecanismo indireto, a Ig monoclonal atua como um autoanticorpo, ativando a VA por meio da inibição de proteínas reguladoras do complemento, sem que haja depósitos de Ig no tecido renal^1,3^. O melhor exemplo de um distúrbio associado à GMSR com deposição ausente ou escassa de Ig monoclonal é a glomerulopatia C3 (GC3), incluindo a doença de depósito denso (DDD) e a glomerulonefrite C3 (GNC3)^1^.

Embora a GC3 seja uma entidade rara, diversos estudos demonstraram uma elevada prevalência de gamopatia monoclonal em pacientes com mais de 50 anos com glomerulopatia C3 (chegando a 65% em algumas séries)^4^, muito superior à prevalência de gamopatia monoclonal na população em geral (4,2%)^5,6^.

A GC3 é um diagnóstico histopatológico, caracterizado por deposição dominante de C3 na coloração por imunofluorescência. A apresentação é variável, incluindo desde hematúria assintomática até glomerulonefrite rapidamente progressiva^7^.

Atualmente, ainda existe uma certa incerteza quanto ao melhor tratamento para pacientes com GNC3 associada à IgM (mIg-C3GN, por sua sigla em inglês). Entretanto, as evidências de algumas séries e relatos de casos sugerem que terapias direcionadas ao mieloma estão associadas a melhores desfechos renais em comparação com terapias imunossupressoras conservadoras ou convencionais^4,8,9^.

Este relato de caso descreve o curso clínico e os desfechos de um paciente com GMSR associada à GNC3 tratado com terapia direcionada ao clone.

## Relato de Caso

Um homem de 61 anos com histórico de insuficiência cardíaca (FEVE 46%) após duas internações por miopericardite com choque cardiogênico em 2018 e 2021. A biópsia miocárdica foi realizada em 2018, mostrando infiltrado linfocitário discreto e inespecífico. Não havia evidência de etiologia imunológica ou neoplásica e presumiu-se um diagnóstico de miopericardite viral. Ele foi diagnosticado com gamopatia monoclonal de significância indeterminada (GMSI) do tipo IgG/kappa em 2021 (pico M de 0,38 g/dL na eletroforese/imunofixação de proteínas séricas e razão de cadeias leves livres kappa/lambda de 5,47).

Em janeiro de 2022, ele apresentou eritrocitúria de 25-50/hpf, proteinúria de 2,5 g/dia (albuminúria de 1700 mg/dia) e função renal preservada (CrS 1,1/ Ur 65 mg/dL, depuração de creatinina 94 mL/min). O diagnóstico de gamopatia monoclonal IgG/kappa foi estabelecido com base nos resultados de imunofixação de soro e urina: proteína M de 0,4 g/dL, nível sérico da cadeia leve kappa de 11,8 mg/L e nível sérico da cadeia leve lambda de 1,16 mg/L, com uma razão correspondente de 10,15. A hemoglobina foi de 12,4 g/dL e o cálcio, de 9,0 mg/dL. Os níveis séricos de imunoglobulina A (IgA), IgG e IgM estavam reduzidos (596, 68 e 21 mg/dL, respectivamente). Seu nível de C3 estava baixo, 74 mg/dL (intervalo normal: 90-180 mg/dL), e seu nível de C4 estava normal (15 mg/dL). Não foi realizado um painel expandido para testes de complemento. Não foram detectadas outras alterações no estudo imunológico (anticorpos anti-GBM, ANAs, anti-dsDNA, ANCAs e crioglobulinas negativos) e as sorologias virais para hepatite B, hepatite C e HIV foram negativas. O aspirado de medula óssea revelou 3,6% de células plasmáticas, sendo 95% delas com um fenótipo anormal.

A biópsia renal mostrou proliferação mesangiocapilar com um padrão membranoproliferativo na microscopia óptica, com hipercelularidade endocapilar, lobulação e membranas basais glomerulares espessadas com contornos duplos; atrofia tubular e fibrose intersticial de 5 e 10%, respectivamente. A imunofluorescência (IF) revelou positividade mesangial e da parede capilar para C3 (2+). C1q foi vestigial, com coloração negativa para imunoglobulinas (IgG, IgA, IgM) ou cadeias leves κ e λ. Na microscopia eletrônica, foram observados depósitos do tipo imune mesangiais, mesangiocapilares e subendoteliais (Figura 1).

**Figura 1:** Biópsia renal. (A e C) Microscopia óptica (HE e tricrômico de Masson, respectivamente) mostrando padrão membranoproliferativo, com hipercelularidade endocapilar, lobulação e membranas basais glomerulares espessadas; (B) Imunofluorescência com coloração para C3; (D) Microscopia eletrônica – depósitos mesangiais, mesangiocapilares e subendoteliais do tipo imune.

Foi feito um diagnóstico de glomerulonefrite C3 associada à gamopatia monoclonal de significância renal, e o paciente iniciou a terapia direcionada ao clone em junho de 2022. Ele recebeu 7 meses de terapia com ciclofosfamida IV 300 mg/m^2^, bortezomibe (1,3 mg/m^2^ semanalmente), e dexametasona (40 mg semanalmente).

Dois meses após o início da quimioterapia, detectou-se uma diminuição da proteína M para 0,2 mg/dL e uma redução da razão de cadeias leves livres kappa/ lambda para 2,2, juntamente com uma melhora da proteinúria para 1,6 g/24 horas. Ao final do tratamento, o paciente obteve resposta hematológica completa, com desaparecimento da proteína monoclonal na eletroforese sérica, normalização da relação kappa/lambda sérica e nenhuma detecção de componentes monoclonais na eletroforese/imunofixação urinária. A proteinúria melhorou ainda mais, para 0,33 g/24 horas, a eritrocitúria desapareceu e a creatinina permaneceu estável em 1,0 mg/dL.

Ele não recebeu terapia de manutenção e permanece estável, com resposta hematológica completa, função renal preservada e proteinúria inferior a 400 mg/24h, 12 meses após a interrupção do tratamento (Figura 2).

**Figura 2:** Respostas hematológicas e renais. *Ciclofosfamida, bortezomibe e dexametasona.

## Discussão

A GMSR é uma condição complexa e desafiadora, caracterizada por danos renais induzidos pela secreção de uma imunoglobulina monoclonal (IgM) nefrotóxica. O padrão da lesão renal é determinado principalmente pelas características estruturais e físico-químicas intrínsecas da proteína monoclonal (imunoglobulinas monoclonais intactas ou cadeias leves de imunoglobulina), e não pela taxa de produção ou características do clone^1,2^. Esse relato de caso destaca a associação entre GMSR e GNC3.

As glomerulopatias C3 são um grupo de doenças renais raras causadas pela desregulação da VA do complemento^7^. A GC3 é caracterizada histopatologicamente pelo acúmulo do componente C3 no tecido renal. Esse achado, na ausência ou quase ausência de depósitos de imunoglobulina, é o único critério diagnóstico. A desregulação da via terminal também pode ocorrer, especialmente na GNC3^7^.

A desregulação da VA pode resultar de alterações adquiridas ou genéticas. Na maioria dos pacientes, a doença é causada por fatores adquiridos, ou seja, autoanticorpos que têm como alvo proteínas reguladoras do sistema complemento. Os mais comuns são os fatores nefríticos C3, que estabilizam a C3 convertase, aumentando sua meia-vida; porém, outros autoanticorpos foram identificados, como os fatores nefríticos C5 (que visam o C3bBbC3b), os fatores nefríticos C4 (voltados para o C4b2a), os autoanticorpos do fator H e do fator B. As causas genéticas são menos frequentes e incluem mutações que resultam em perda de função nos genes responsáveis por proteínas reguladoras ou em ganho de função em proteínas ativadoras^7,10,11^.

Mais recentemente, foi descrita uma associação entre gamopatia monoclonal e GC3^4,7^. A fisiopatologia da mIg-C3G ainda está sob investigação, mas evidências sugerem uma associação entre a IgM e a ativação inadequada da VA: as cadeias leves monoclonais podem atuar como autoanticorpos contra o fator H, resultando na diminuição da atividade do fator H, e a própria IgM pode atuar como fator nefrítico C3^7,10^. No entanto, a imunoglobulina monoclonal nem sempre apresenta atividade de anticorpo contra as proteínas reguladoras do complemento e acredita-se que ative a via alternativa por meio de outros mecanismos^4,10,12^. Anormalidades genéticas em genes de complemento raramente são identificadas na mIg-C3G^4,7,12^.

A apresentação clínica da glomerulopatia C3 varia desde hematúria assintomática e proteinúria de graus variáveis, incluindo a faixa nefrótica, até uma apresentação aguda com síndrome nefrítica, injúria renal aguda ou glomerulonefrite rapidamente progressiva^7^. Os níveis séricos de C3 são baixos na maioria dos pacientes, enquanto os níveis de C4 são geralmente normais; níveis séricos elevados de sC5b-9 podem estar presentes^4,7^. Pacientes com mIg-C3G são normalmente mais velhos em comparação com pacientes com GC3 sem Ig monoclonal e tendem a exibir níveis mais elevados de creatinina sérica e proteinúria na apresentação^4^.

A biópsia renal é obrigatória para estabelecer o diagnóstico^7^. Todos os pacientes com GMSI com suspeita de envolvimento renal, ou seja, suspeita de GMSR (por exemplo, apresentando proteinúria, hematúria e/ou comprometimento inexplicável da função renal), devem ser submetidos a uma biópsia renal, uma vez que o diagnóstico renal pode alterar drasticamente o panorama terapêutico e prognóstico da doença. Pelo mesmo motivo, a biópsia renal também é recomendada em pacientes com GMSI e outros fatores de risco conhecidos para doença renal crônica que apresentem um curso clínico atípico^1^. O presente caso exemplifica o impacto significativo de um diagnóstico oportuno, aliado a um tratamento eficaz, no prognóstico renal e no curso da doença.

Na microscopia óptica, a GC3 geralmente apresenta um padrão membranoproliferativo, mas também pode exibir proliferação mesangial ou endocapilar. Podem ser observados padrões exsudativos, crescênticos e esclerosantes. Os dois principais subtipos de glomerulopatia C3, DDD e GNC3, são diferenciados por sua aparência ultraestrutural: depósitos mesangiais, subepiteliais e subendoteliais difusos e moderadamente densos em elétrons são observados na glomerulonefrite C3, enquanto depósitos intramembranosos altamente densos em elétrons, em forma de “salsicha” e depósitos mesangiais nodulares arredondados são observados na doença de depósito denso. subepiteliais podem ocorrer em ambos os subtipos^7,11^. A microscopia de imunofluorescência, essencial para o diagnóstico, mostra coloração para C3 com pelo menos o dobro da intensidade de qualquer outro imunoreagente e, normalmente, é negativa para Ig (cadeias pesadas ou leves)^7,13^.

A GNC3 é a forma mais comum de glomerulopatia C3 com gamopatia monoclonal. É importante ressaltar que uma minoria de pacientes (5-10%) com gamopatia monoclonal e achados na imunofluorescência padrão consistentes com GNC3 terá, na verdade, uma glomerulonefrite membranoproliferativa com depósitos monoclonais mascarados. Esses pacientes precisam de estudos adicionais de imunofluorescência a serem realizados em tecido embebido em parafina e digerido por protease para identificação da imunoglobulina monoclonal nos depósitos^1^. A disponibilidade limitada dessas técnicas auxiliares em nosso país inviabilizou sua utilização neste caso.

Além disso, a biópsia renal fornece informações prognósticas valiosas^7,14^. O índice histológico de GC3 da *Columbia University*, validado em 2017, avalia a atividade e cronicidade da doença com base em características histológicas, incluindo hipercelularidade mesangial, proliferação endocapilar, morfologia membranoproliferativa, crescentes celulares e fibrocelulares, necrose fibrinoide e inflamação intersticial (em uma escala de 0 a 3). As características avaliadas para o escore de cronicidade incluem glomeruloesclerose, atrofia tubular, fibrose intersticial (cada uma em uma escala de 0 a 3) e arteriosclerose (em uma escala de 0 a 1). No estudo de Bomback et al.^14^, a taxa de filtração glomerular estimada no momento do diagnóstico, o percentual de atrofia tubular e o percentual de fibrose intersticial foram os preditores independentes mais fortes da progressão para a perda da função renal. Em um estudo mais recente, embora menor, de Caravaca-Fontán et al.^12^, o índice histológico de GC3 foi aplicado a 23 pacientes com GC3 e gamopatia monoclonal, com escores de cronicidade mais elevados estando associados a piores desfechos renais. Notavelmente, escores mais elevados de cronicidade no momento da biópsia renal podem ser a expressão de um diagnóstico tardio nesta população com idade mais avançada com mIg-C3G^12^. Assim, embora desafiador, um diagnóstico precoce, anterior ao desenvolvimento de lesões crônicas, é um fator prognóstico crucial, permitindo que as intervenções terapêuticas sejam eficazes no nível do parênquima renal.

Muitos pacientes mais velhos, especialmente aqueles com idade ≥50 anos que apresentam glomerulopatia C3, terão gamopatia monoclonal, indicativa de GMSR^4,12,15^. Em uma grande coorte da *Mayo Clinic,* a gamopatia monoclonal foi identificada em apenas 8 de 52 (15%) pacientes <50 anos de idade, em comparação com 28 de 43 (65%) pacientes >50 anos de idade^15^. Assim, todos os pacientes com idade ≥50 anos com GC3 devem ser triados para paraproteínas por meio de eletroforese de proteínas séricas, imunofixação e avaliação de cadeias leves livres séricas. O diagnóstico de gamopatia monoclonal exige uma avaliação adicional com biópsia da medula óssea para identificar a população clonal responsável pela produção de IgM^1,7^. A doença hematológica subjacente mais comum em pacientes com mIg-G3C é a GMSI/GMSR, sendo IgG/κ o isótipo de IgM mais comum^2,4^.

A opinião de especialistas sugere que pacientes com glomerulopatia C3 devem ser submetidos a uma avaliação abrangente do complemento, incluindo a avaliação geral da atividade do complemento, medições dos níveis séricos das proteínas do complemento e seus produtos de clivagem, além da triagem de autoanticorpos^7^. Testes genéticos também devem ser considerados, embora seu valor preciso no cenário clínico de pacientes com mIg-C3G ainda precise ser determinado^7,12^. Essa é uma limitação do nosso estudo, uma vez que nem a triagem para autoanticorpos nem os estudos genéticos do complemento foram realizados. No entanto, a falta dessas informações não parece ter afetado significativamente a avaliação diagnóstica e prognóstica de nosso paciente, conforme evidenciado por sua excelente resposta ao tratamento.

O melhor tratamento para GNC3 associada à GMSR ainda não foi estabelecido^7^. No entanto, existem evidências crescentes que sugerem a superioridade das terapias direcionadas ao clone em termos de sobrevida renal em comparação com a imunossupressão convencional ou o manejo conservador, destacando a correlação entre a redução de IgM e melhores desfechos renais^4,9,12^. Chauvet et al.^8^ constataram que os pacientes que receberam quimioterapia, incluindo bortezomibe, obtiveram melhor resposta renal do que aqueles que receberam terapia conservadora/imunossupressora. Nesse estudo, a obtenção de uma resposta hematológica esteve significativamente associada a uma maior sobrevida renal^8^. Esses resultados foram posteriormente reproduzidos por alguns estudos observacionais, confirmando a superioridade terapêutica das terapias direcionadas ao clone em termos de sobrevida renal e a correlação da resposta hematológica com a melhoria da proteinúria e dos desfechos renais^4,9,12,13^. Com base nesses achados, agentes antimieloma como bortezomibe, lenalidomida ou até mesmo daratumumabe devem ser considerados como terapia de primeira linha para mIg-C3G até que mais dados estejam disponíveis. Na prática clínica normal, às vezes é difícil incentivar os hematologistas a realizarem esse tipo de tratamento na ausência de um processo neoplásico ou de evidências claras de deposição de IgM no tecido renal, o que representa um desafio no manejo desses pacientes. O papel potencial das novas terapias direcionadas ao complemento como tratamento adjuvante em pacientes selecionados com mIg-C3G ainda precisa ser avaliado^7^.

Da mesma forma, o tratamento ideal para a GC3 sem IgM não foi estabelecido. A maioria desses pacientes é tratada com imunossupressão convencional (corticosteroides isolados ou corticosteroides combinados com outros medicamentos), com resultados variados^7^. Em um estudo de Ravindran et al.^4^, não houve diferença significativa na sobrevida renal entre pacientes com mIg-C3G e pacientes com GC3 sem IgM. No contexto específico da mIg-C3G, a terapia direcionada ao clone pode resultar em melhora da sobrevida renal^4,7,9,13^.

Os dados limitados sobre mIg-C3G no transplante renal apontam para um alto risco de recidiva da doença em receptores de aloenxertos^7^. Além disso, receptores de transplante com C3G-mIg parecem apresentar desfechos renais ruins, apesar da obtenção de uma resposta hematológica em alguns casos^12^. Em pacientes mais jovens e selecionados que são candidatos ao transplante, o transplante autólogo de células-tronco pode ser considerado como uma terapia complementar, ajudando a alcançar uma resposta hematológica mais profunda e sustentada, ou até mesmo completa, o que é essencial para reduzir o risco de recidiva, principalmente após o transplante renal^16^.

A abordagem terapêutica adotada nesse caso foi a terapia direcionada ao clone. Notavelmente, o paciente obteve uma resposta hematológica completa, com desaparecimento do pico M na eletroforese sérica e normalização da razão de cadeias leves livres kappa/lambda no soro. O sucesso da terapia direcionada ao clone neste caso destaca a importância de estratégias de tratamento personalizadas na GC3 associada à GMSR, em concordância com as evidências mais recentes^4,8,9,12,13^. Além disso, foi estabelecido um diagnóstico precoce, com poucos sinais de cronicidade na biópsia renal. Isso, aliado à eficácia da terapia direcionada ao clone, influenciou de forma significativa o prognóstico renal do paciente. O acompanhamento de longo prazo revelou remissão sustentada da condição hematológica e da proteinúria, mantendo a função renal preservada, reforçando que a obtenção de uma resposta hematológica completa ou profunda resulta em melhores desfechos renais.

## Conclusões

Em conclusão, este caso ressalta a relação complexa entre GMSR e GNC3, destacando a importância de estratégias de tratamento personalizadas. O paciente apresentou uma resposta excepcional ao tratamento, demonstrando a importância do diagnóstico precoce e da terapia eficaz direcionada ao clone na melhora do prognóstico renal. A remissão de longo prazo observada tanto nos parâmetros hematológicos quanto renais enfatiza a importância da terapia direcionada ao clone e a necessidade de acompanhamento constante no manejo dos casos de GC3 associados à GMSR.
